# Cilostazol Induces eNOS and TM Expression via Activation with Sirtuin 1/Krüppel-like Factor 2 Pathway in Endothelial Cells

**DOI:** 10.3390/ijms221910287

**Published:** 2021-09-24

**Authors:** Chih-Hsien Wu, Yi-Lin Chiu, Chung-Yueh Hsieh, Guo-Shiang Tsung, Lian-Shan Wu, Cheng-Chung Cheng, Tsung-Neng Tsai

**Affiliations:** 1 National Defense Medical Center, Department of Biochemistry, Taipei 114201, Taiwan; claudia_csmu13@hotmail.com (C.-H.W.); yc566@georgetown.edu (Y.-L.C.); 2 Department of Internal Medicine, Division of Cardiology, Tri-Service General Hospital, National Defense Medical Center, Taipei 114202, Taiwan; chengcc@mail.ndmctsgh.edu.tw; 3 Department of Internal Medicine, Division of Cardiology, Taoyuan Armed Forces General Hospital, Taoyuan City 325208, Taiwan; hcy020162@gmail.com (C.-Y.H.); shangz0216@gmail.com (G.-S.T.); 4 Department of Internal Medicine, Division of Cardiology, Hualien Armed Forces General Hospital, Hualien County 325208, Taiwan; s3505476@yahoo.com.tw

**Keywords:** cilostazol, endothelial cells, Krüppel-like factor 2, endothelial nitric oxide synthase, nitric oxide, thrombomodulin

## Abstract

Cilostazol was suggested to be beneficial to retard in-stent atherosclerosis and prevent stent thrombosis. However, the mechanisms responsible for the beneficial effects of cilostazol are not fully understood. In this study, we attempted to verify the mechanism of the antithrombotic effect of cilostazol. Human umbilical vein endothelial cells (HUVECs) were cultured with various concentrations of cilostazol to verify its impact on endothelial cells. KLF2, silent information regulator transcript-1 (SIRT1), endothelial nitric oxide synthase (eNOS), and endothelial thrombomodulin (TM) expression levels were examined. We found cilostazol significantly activated KLF2 expression and KLF2-related endothelial function, including eNOS activation, Nitric oxide (NO) production, and TM secretion. The activation was regulated by SIRT1, which was also stimulated by cilostazol. These findings suggest that cilostazol may be capable of an antithrombotic and vasculoprotective effect in endothelial cells.

## 1. Introduction

Endothelial cells, the lining in the lumen of blood vessels, play various roles in the physiological and pathological processes of blood circulation. One of the important functions of endothelial cells is the regulation of hemostasis and fibrinolysis activity within the bloodstream. Moreover, the endothelial cells expression and secretion of several critical anticoagulation factors, such as nitric oxide (NO) and thrombomodulin (TM), maintain the fluidity of blood within the vessel and prevent thrombus formation. Krüppel-like factor 2 (KLF2), one of the transcription factors of the zinc finger family, was shown to not only involve the differentiation of cells and development of tissue but also to impact endothelial function [1,2,3,4]. Dekker et al. demonstrated that the overexpression of KLF2 could stimulate the expression of TM and endothelial nitric oxide synthase (eNOS) and inhibit plasminogen activator inhibitor type-1 on endothelial cells [5]. Additionally, some studies reported that stimulated KLF2 using shear stress can regulate the hemostasis function and anti-inflammatory effects as well as enhance NO production of endothelial cells [4,6,7]; these suggest the vasculoprotective role of KLF2. According to previous studies, several small molecular compounds, such as statin and resveratrol, showed the vascular effects of increased KLF2 expression through increased eNOS activity and enhanced NO production [8,9].

Cilostazol, a vasodilatory agent, increases intracellular cAMP concentrations and causes vascular dilation. It had been used as the first-line drug to treat claudication of peripheral arterial occlusive disease. Using in vitro studies and clinical observation, recent reports showed that cilostazol may provide antiplatelet and antithrombotic effects [10,11,12]. Additionally, the effect of vasodilation and cilostazol has been shown to prevent endothelial cell apoptosis [13,14] and monocyte adhesion [15,16] as well as to stimulate angiogenic factors [17]. However, the mechanisms of these effects are not fully understood. In this study, we investigated the impact of cilostazol on endothelial cells related to thrombus formation, such as stimulating eNOS expression, NO production, and TM activity via KLF2 activation. Additionally, the potential molecular mechanisms that act on this process were explored.

## 2. Results

### 2.1. Cilostazol Showed a Negative Correlation with Thrombosis-Related Gene Sets Investigated in GSE19151

**Figure 1:**
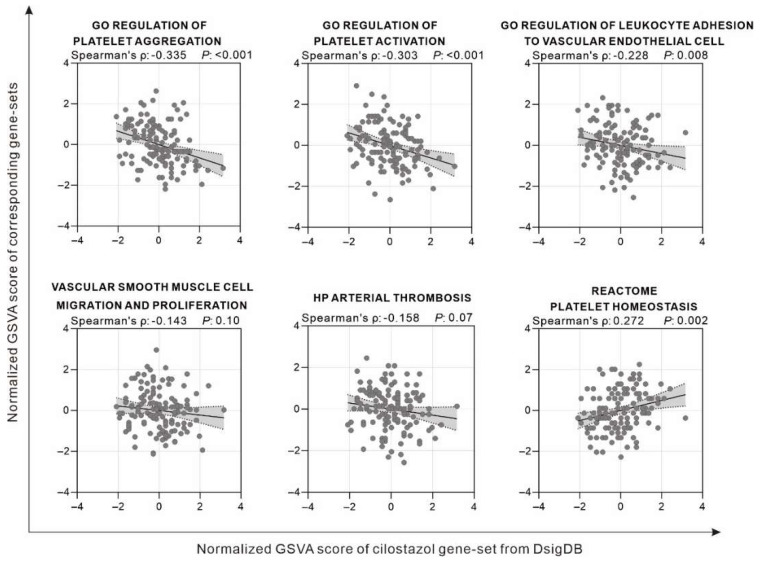
Association of GSVA score of cilostazol gene-set with various thrombosis-related gene sets investigated in GSE19151.

The thrombus formation could relate to the platelet activation, endothelial cell dysfunction, and smooth muscle cell migration that are associated with the NO and TM secretion in the endothelial cells. The previous study had shown that resveratrol activates *SIRT1* and *KLF2* gene expression, conferring eNOS and TM expression in endothelial cells [8]. To verify the impact of cilostazol on cardiovascular system-related genes in clinical patients, the GSVA strategy in combination with the cilostazol gene set provided by DsigDB was used to analyze the clinical thrombosis patient samples provided by GSE19151. The results showed that the GSVA scores of cilostazol-affected genes were positively correlated with “PLATELET HOMEOSTASIS” and negatively correlated with “PLATELET AGGREGATION” and “PLATELET ACTIVATION”, suggesting that cilostazol-inhibited platelet activation and coagulation were in accordance with our speculation in cellular experimental observations below. In addition, the GSVA score of cilostazol was negatively correlated with the scores of “Vascular Smooth Muscle Cell Migration and Proliferation”, “ARTERIAL THROMBOSIS”, and “LEUKOCYTE ADHESION”, suggesting that cilostazol processing in the clinic may affect the cardiovascular system via the SIRT1–KLF2–TM/eNOS axis (Figure 1).

### 2.2. Cilostazol Upregulates KLF2 Expression in Endothelial Cells

**Figure 2:**
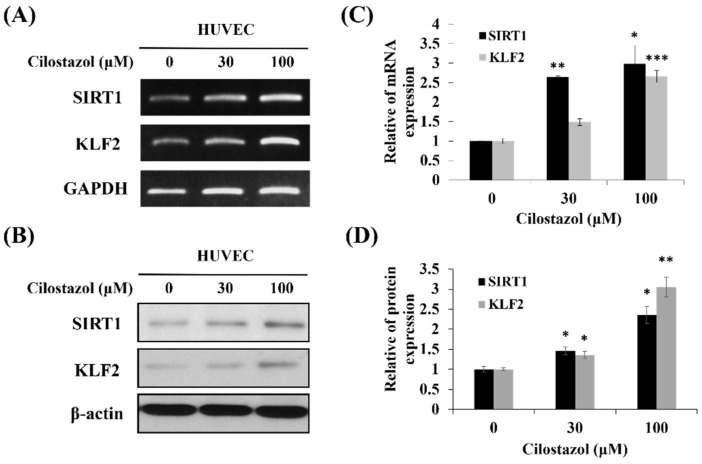
Cilostazol upregulates the expression of Krüppel-like factor 2 (KLF2). The HUVECs were incubated with 0, 30, and 100 μM cilostazol for 24 h. The mRNA expression of KLF2 and silent information regulator transcript-1 (SIRT1) in endothelial cells was assessed using (**A**) PCR and (**B**) quantitative real-time PCR, respectively. Additionally, protein expression was verified using (**C**,**D**) Western blot. Data are expressed as mean ± SEM. * *p* < 0.05, ** *p* < 0.01 and *** *p* < 0.001 vs. control.

It was reported that the HUVECs were observed to be elongated and spindle-shaped after exposure to shear stress [18], which can induce KLF2 expression [19]. KLF2 activation by shear stress involved the cytoskeleton redistribution and contributed to stress fiber formation in endothelial cells [20]. To verify whether cilostazol upregulates KLF2 expression, the mRNA and protein levels of the HUVECs were analyzed using real-time PCR and Western blot. After the HUVECs were treated with cilostazol for 24 h, the KLF2 expression increased dose-dependently in RNA and protein levels (Figure 2).

### 2.3. Cilostazol Stimulates eNOS and TM Expression, and, Subsequently, Their Bioavailability

**Figure 3:**
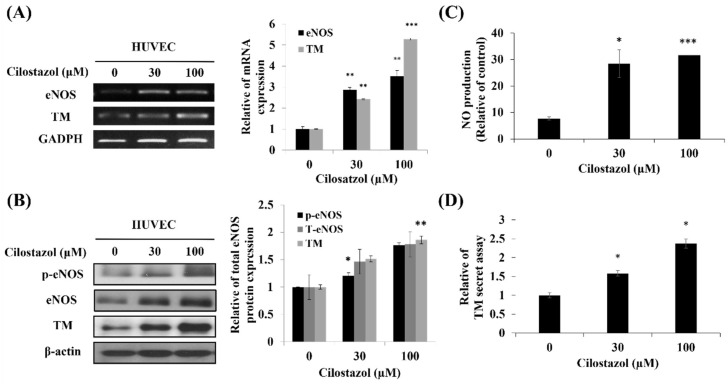
Cilostazol increases endothelial nitric oxide synthase (eNOS) expression and thrombomodulin (TM) at the RNA and protein levels. The HUVECs were incubated with cilostazol (0, 30, or 100 µM) for 24 h. The expression of eNOS and TM on mRNA and *p*-eNOS, total eNOS, and TM on protein expression levels was assessed using (**A**) PCR and (**B**) Western blot, respectively. (**C**) The nitric oxide (NO) production and (**D**) TM secretion was detected using flow cytometry with 4,5-diaminofluorescein and ELISA, respectively. Data are expressed as mean ± SD of experiments conducted in triplicate. * *p* < 0.05, ** *p* < 0.01, and *** *p* < 0.001 compared to the untreated group.

Activated KLF2 plays a critical role in stimulating eNOS and TM activity as well as subsequent NO production when the endothelium is exposed to shear stress [21]. To explore eNOS and TM expression after cilostazol treatment, the HUVECs were treated with 0, 30, and 100 µM cilostazol for 24 h. RT-PCR and Western blot were used to evaluate the effect of cilostazol on eNOS and TM expression. According to the result, in RNA and protein levels, cilostazol enhanced either eNOS or TM expression in a dose-dependent manner (Figure 3A,B). Functional assays also presented that cilostazol increased NO production and TM activity (Figure 3C,D).

### 2.4. KLF2 Expression Is Critical for eNOS and TM Induction in Cilostazol-Treated HUVECs

**Figure 4:**
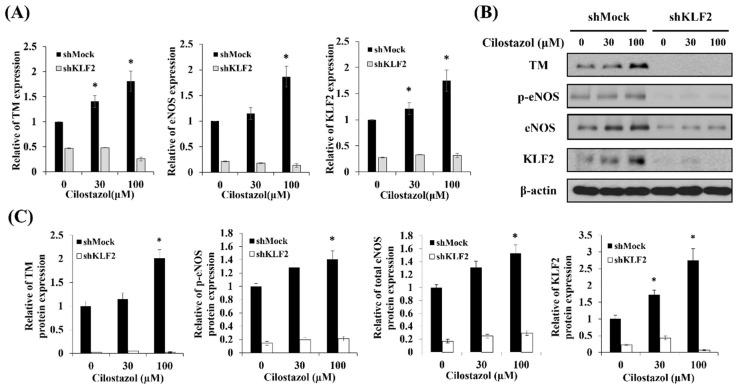
Knockdown of *KLF2* decreases eNOS and TM expression in cilostazol-treated endothelial cells. Silence *KLF2* expression by transfecting HUVECs with short hairpin KLF2 (shKLF2) and a mock vector (shMock) was used as a control. Transfected cells were incubated with 0, 30, or 100 µM cilostazol for 24 h. (**A**) Real-time PCR and (**B**) Western blot were used to identify *p*-eNOS, total eNOS, and TM in either RNA or protein expression, respectively. (**C**) Reflective densitometry of Western blot. Data are presented as mean ± SEM, *n* = 3. * *p* < 0.05 compared to the untreated group.

**Figure 5:**
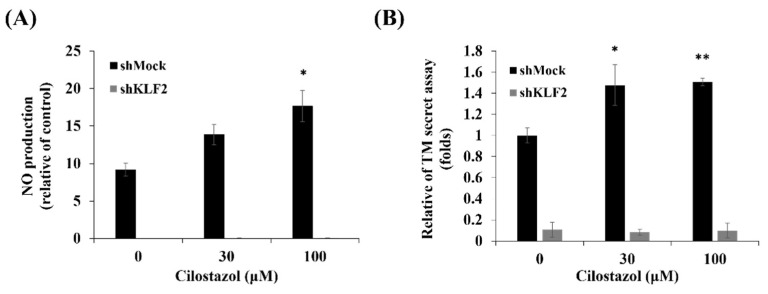
The NO production and TM secretion on cilostazol-treated shKLF2 cells. The HUVECs, shMock, or shKLF2 were treated with cilostazol for 24 h. (**A**) The NO production was measured by 4,5-diaminofluorescein using flow cytometry. (**B**) TM secretion was assessed by ELISA. Data are presented as mean ± SEM, *n* = 3. * *p* < 0.05 and ** *p* < 0.01 compared to the untreated group.

To verify the role of KLF2 in eNOS and TM expression in the cilostazol-treated HUVECs, shRNA was used to knockdown the *KLF2* gene expression. After being transfected with shMock or shKLF2, the HUVECs were treated with cilostazol for 24 h. KLF2 silenced cells had lower eNOS, TM mRNA, and protein levels, even when incubated with cilostazol (Figure 4). To further analyze the effects of cilostazol on NO production and TM activity in shKLF2 cells, the cells’ expression was examined via flow cytometry and ELISA. The results showed that shKLF2 downregulated the cilostazol-induced NO production and TM activity (Figure 5). It suggested that KLF2 is critical for cilostazol-induced eNOS, TM expression, NO production, and TM activity.

### 2.5. Cilostazol Induces KLF2 via SIRT1 Activation

**Figure 6:**
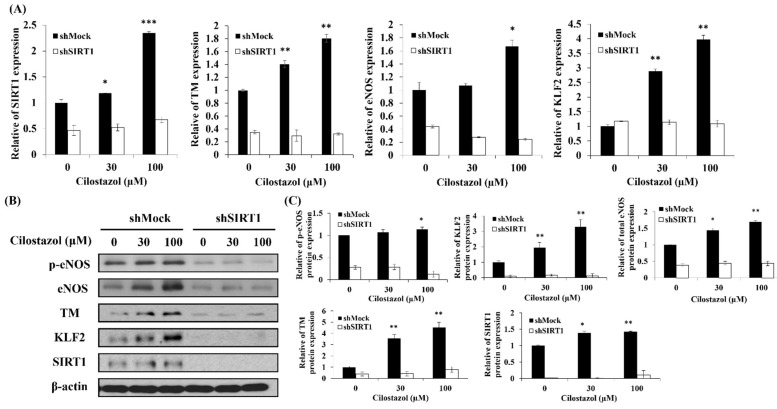
SIRT1 as a regulator of KLF2 in the cilostazol-treated HUVECs. HUVECs, shMock, or shSIRT1 were cultured with 0, 30, and 100 μM cilostazol. After 24 h, the cells were collected. The SIRT1 mRNA expression was identified using either (**A**) quantitative real-time PCR or (**B**) the protein expression was determined using Western blot. (**C**) Reflective densitometry of Western blot. Data are expressed as mean ± SEM, *n* = 3. * *p* < 0.05, ** *p* < 0.01 and *** *p* < 0.001 compared to the untreated group [27].

**Figure 7:**
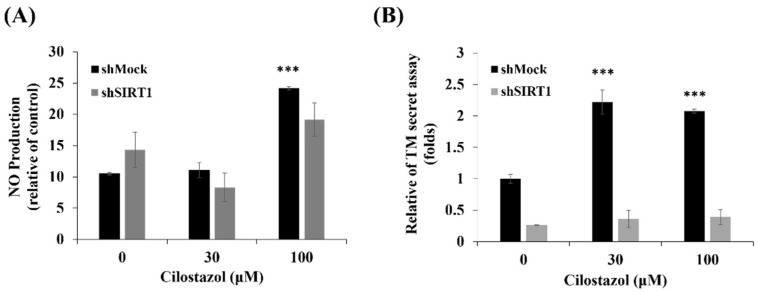
The NO production and TM secretion on cilostazol-treated shSIRT1 cells. HUVECs, shMock, or shSIRT1 were cultured with 0, 30, and 100 μM cilostazol for 24 h. (**A**) The NO production of cells was stained with 4,5-diaminofluorescein and analyzed using flow cytometry. (**B**) The TM secretion was measured using ELISA. Data are presented as mean ± SEM, *n* = 3. *** *p* < 0.001 compared to the untreated group.

**Figure A1:**
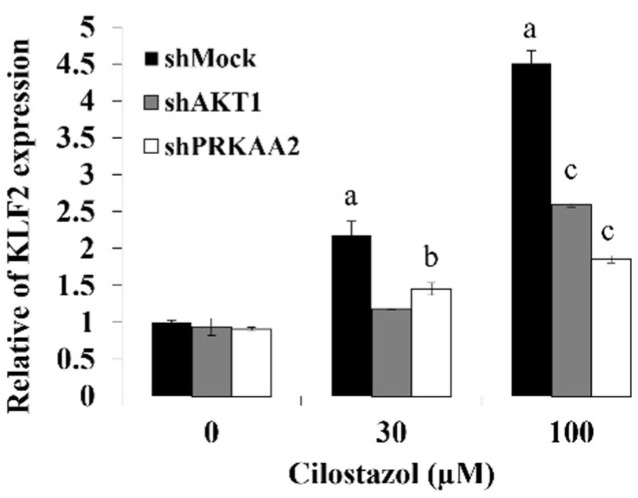
Cilostazol upregulates KLF2 partially through PRKAA2 or AKT1 pathways. The HUVECs were transfected with shRNA, which blocked AKT1 or PRKAA2 expression. shMock was used as the vector control. After transfection, cells were cultured with 0, 30, and 100 μM cilostazol for 24 h. Then, cells were collected, and KLF2 mRNA expression was assessed using quantitative real-time PCR. Data are expressed as mean ± SEM, *n* = 3. a, *p* < 0.05 compared to shMock 0 μM cilostazol; b, *p* < 0.05 compared to shMock 30 μM cilostazol; c, *p* < 0.05 compared to shMock 100 μM cilostazol.

SIRT1, a deacetylase that contributes to cellular longevity and metabolic homeostasis, is suggested to be related to KLF2 expression in endothelial cells [8,22]. We observed that cilostazol-treated HUVECs increased SIRT1 expression in a dose-dependent manner (Figure 2A). Silenced *SIRT1* expression blocked the cilostazol-mediated KLF2, eNOS, and TM expression (Figure 6). Furthermore, the NO production and TM activity were attuned in *SIRT1* silenced cells despite cilostazol treatment (Figure 7). According to our data, SIRT1 acts as an upstream regulator of cilostazol-stimulated KLF2 activation on HUVECs. Previous reports have shown that activated PRKAA2 (protein kinase AMP-activated catalytic subunit alpha 2; AMP-activated kinase, AMPK) and AKT1 by shear stress increase KLF2 expression in endothelial cells [23,24]. Since both pathways have also been reported to be activated after cilostazol treatment [25,26], we investigated whether both kinases are involved in the cilostazol-mediated KLF2 induction by silencing them with shRNA. The results showed that shPRKAA2 and shAKT1 downregulate the cilostazol-induced KLF2 expression (Figure A1). It suggested that cilostazol increased KLF2 partially through the PRKAA2 and AKT1 pathways.

## 3. Discussion

Stent thrombosis, thrombotic occlusion of a stent, could lead to lethal acute coronary syndrome or restenosis-related angina [28]. Cilostazol, a phosphodiesterase 3 inhibitor, has been widely used as an antiplatelet and vasodilatory agent to treat claudication caused by peripheral artery disease. It was prescribed for patients undergoing unresponsive dual antiplatelet therapy after percutaneous coronary intervention (PCI) [29]. Cleanthis, M. et al. demonstrated that the combination of aspirin with cilostazol could suppress platelet activation when patients exercised by using free platelet-counting aggregation and flow cytometry for surface markers of platelet activation and soluble P-selectin assay [30]. Geng et al. reported that cilostazol-based triple antiplatelet therapy after coronary stenting could reduce the risk of cardiovascular thrombotic events [31]. Moreover, Jeon et al. compared triple antiplatelet therapy to dual therapy after implanting drug-eluting stents. According to their findings, adding cilostazol to aspirin and clopidogrel may be more effective in preventing stent thrombosis during the first six months [32]. The formation of stent thrombosis is related to platelet function activation, endothelial cell dysfunction, and smooth muscle cell migration. To evaluate the effect of cilostazol on cardiovascular system-related genes, the GSVA strategy in combination with the cilostazol gene set provided by DsigDB were used to analyze the clinical thrombosis patient samples provided by GSE19151. We found the cilostazol-affected genes were related to the inhibition of platelet activation. Moreover, the GSVA score of cilostazol was negatively correlated with the scores of “ARTERIAL THROMBOSIS”, “LEUKOCYTE ADHESION”, and “Vascular Smooth Muscle Cell Migration and Proliferation”. It suggested that cilostazol may affect endothelial cell dysfunction and smooth muscle cell migration related to thrombosis formation. These data imply the antithrombotic effect of cilostazol in thrombosis.

While our data revealed cilostazol has an impact on thrombus formation, the mechanisms are still not fully investigated. TM, an integral membrane protein present on the surface of endothelial cells, participates in hemostasis. As a cofactor for thrombin, the TM-bound thrombin not only facilitates the activation of protein C but also inhibits fibrinolysis. Activated protein C inhibits the activity of potential procoagulant cofactors, factor Va and factor VIIIa, which reduced the formation of thrombin and, subsequently, blood clots. Moreover, in the process of thrombus formation, eNOS activation and, subsequently, NO production has been shown to inhibit platelet aggregation. In this study, we discovered that cilostazol can increase TM and eNOS expression as well as their bioavailability in vitro. The cilostazol-induced TM and eNOS expression may be activated through KLF2. Silencing *KLF2* expression attuned eNOS and TM expression, even when the HUVECs were incubated with cilostazol. Our findings suggest that KLF2 is critical for cilostazol-induced eNOS activation as well as TM and NO production, which may involve the antithrombotic effect of cilostazol.

**Figure 8:**
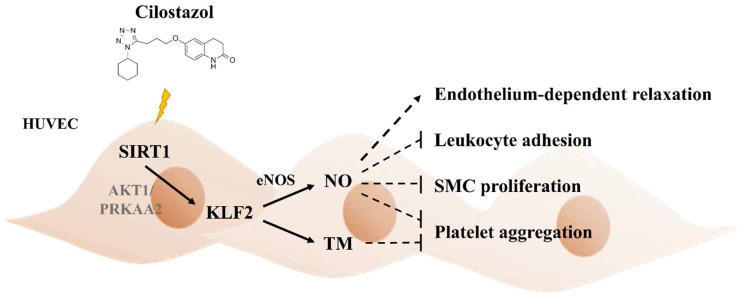
Illustration of the proposed molecular mechanism by cilostazol-treated HUVECs. The suggested working model for the cilostazol induction of KLF2. As delineated, cilostazol activates KLF2 through SIRT1 induction via AKT1 or PRKAA2 pathways, followed by stimulating eNOS and TM expression in HUVECs. Lines with arrowheads indicate activation. SMC, smooth muscle cells.

SIRT1, an NAD^+^-dependent histone deacetylase, involves cell cycle regulation, senescence, metabolism, and longevity. In numerous studies, the role of SIRT1 in endothelial cell function appears to coordinate with KLF2 expression [8,33]. Wu et al. reported particulate matter air pollution exposure decreased lung KLF2 and TM expression in mice models, which could be reversed via *Sirt1*-gene delivery [33]. Gracia-Sancho et al. demonstrated that resveratrol activates SIRT1 and induces KLF2 expression, conferring an increase in eNOS expression in cultured endothelial cells [8]. To further explore the relationship between *SIRT1* and *KLF2* gene expression in cilostazol-treated endothelial cells, the cells were treated with different concentrations of cilostazol. We discovered that not only does cilostazol increase the KLF2 expression in a dose-dependent manner, but it does so for SIRT1, as well. Silencing *SIRT1* expression using the shSIRT1 vector aborted the expression of KLF2, eNOS, and TM in RNA and protein levels, followed by eNOS and TM stimulation. Our result implies that KLF2 induction and subsequent NO and TM production using cilostazol could thwart SIRT1 activation (Figure 8). As compared to the Ota et al. study, we explored more details surrounding the molecular mechanism and function of cilostazol [34].

In addition, previous studies revealed that shear-stress-enhanced KLF2 expression mediated a mitogen-activated protein kinase 5/extracellular signal-regulated kinase 5/myocyte enhancer factor 2 (MEK5/ERK5/MEF2) signaling pathway [35]. Moreover, shear stress enhanced the eNOS-mediated AKT and AMPK pathways [23,36,37], which was observed in cilostazol-treated endothelial cells [26]. On the basis of these findings, we investigated the involvement of AKT and PRKAA2 (AMPK) in the regulation of KLF2 using cilostazol. Surprisingly, our data showed that silencing *AKT* and *PRKAA2* decreased cilostazol-mediated KLF2 expression. This implies that cilostazol might partially stimulate KLF2 via AKT or PRKAA2 pathways.

Several studies have revealed that linear shear stress can lead to the redistribution of the cytoskeleton via KLF2 activation [20,38]. Dekker et al. showed that prolonged KLF2 overexpression could promote cytoskeleton redistribution and cellular morphological change in human endothelial cells, unrelated to shear stress exposure [5]. Our previous study showed that GbE-treated HUVEC could alter cellular morphology via KLF2 activation [39]. In this study, we found that the HUVECs had alterations from a typical cobblestone shape to a spindle form after being incubated with cilostazol. It suggests that cilostazol-induced KLF2 activation might enhance not only eNOS and TM expression but also cytoskeleton rearrangement. However, the mechanisms by which cilostazol impacted the cytoskeleton of endothelial cells were not identified in this study. Further investigations should be conducted to obtain the possible pathways involved.

## 4. Materials and Methods

### 4.1. Gene Expression Profiling and GSEA

Clinical cardiovascular patient sample data were obtained from NCBI’s Gene Expression Omnibus GSE19151 [40]. Cilostazol-related gene sets were downloaded from DsigDB, mainly obtained using the Biomedical Object Search System (BOSS) combined with a text-mining approach [41]. The gene sets “GO REGULATION OF PLATELET AGGREGATION”, “GO REGULATION OF PLATELET ACTIVATION”, “GO REGULATION OF LEUKOCYTE ADHESION TO VASCULAR ENDOTHELIAL CELL” were extracted from the Gene Ontology Molecular Signature Database [42,43]. “VASCULAR SMOOTH MUSCLE CELL MIGRATION AND PROLIFERATION” was extracted from Elsevier Pathway Collection in Enrichr [44]. “HP ARTERIAL THROMBOSIS” was from Human Phenotype Ontology [45], and “REACTOME PLATELET HOMEOSTASIS” was from the Reactome Project [46]. Briefly, the GSE19151 data were calculated using the GSVA package in R, combining the above gene sets with default settings. The results were pre-normalized and analyzed using Spearman’s correlation; then they were plotted with GraphPAD Prism^®^ software, version 6 (GraphPad Software Inc., San Diego, CA, USA).

Human umbilical vein endothelial cells (HUVECs) were obtained from Lonza (Walkersville, MD, USA). The cells were cultured using a sterile endothelial growth medium (EGM-2, Lonza, Walkersville, MD, USA) and maintained at 37 °C in a 5% CO_2_ environment throughout the experiment. HUVECs at 80–90% confluence were used in the experiment and treated with either a vehicle (dimethyl sulfoxide; Sigma-Aldrich, St. Louis, MO, USA) or various concentrations of cilostazol (30 and 100 µM) for 24 h.

### 4.2. Cilostazol Preparation and Storage

Cilostazol was kindly provided by Yung Shin Pharmaceutical Industrial Co., Ltd. (Taiwan). Briefly, the provided cilostazol was used for the preparation of the stock solution. One gram of cilostazol was dissolved into 10 mL sterile phosphate-buffered saline (PBS; Invitrogen, Grand Island, NY, USA) to make a stock solution with a concentration of 100 mg/mL (equivalent to 270.6 mM). Then, the cilostazol suspension was filtered twice through a 0.22-µm standard filter (Pall Corporation, Port Washington, NY, USA). The filtrated solution was frozen in aliquots at −20 °C.

### 4.3. Reverse Transcription Polymerase Chain Reaction (RT-PCR) and qRT-PCR

Total RNA was isolated from the HUVECs in 3.5 cm culture dishes treated with cilostazol in the presence of TRIzol reagent (15596026; Invitrogen, Carlsbad, CA, USA), according to the manufacturer’s instructions. RNA concentrations were spectrophotometrically determined at 260 nm. First-strand cDNA synthesis was conducted with 2 μg total RNA using random hexamers as primers in a final volume of 20 μL (100 ng/μL) random hexamers (Protech Technology, Taipei, Taiwan), 1 mM dNTPs (Protech Technology, Taipei, Taiwan), 1 U/μL RNase inhibitor (Promega, Madison, WI, USA), and 5 U/μL Moloney Murine Leukemia Virus Reverse Transcriptase (Protech Technology, Taipei, Taiwan). The reaction was conducted at 42 °C for 90 min. cDNAs encoding glyceraldehyde 3-phosphate dehydrogenase (GAPDH), silent information regulator transcript-1 (SIRT1), KLF2, TM, and eNOS were amplified from the 2 μL cDNA reaction mixture using specific gene primers. The primers were as follows: GAPDH, 5′-CCTCCCGCTTCGCTCTCTG-3′ (forward), and 5′-GCGCCCAATACGACCAAATC-3′ (reverse); KLF2, 5′-CTACACCAAGA GTTCGCATCTG-3′ (forward), and 5′-CCGTGTGCTTTCGGTAGTG-3′ (reverse); eNOS, 5′-TGATGGCGAAGCGAGTGAAG-3′ (forward), and 5′-ACTCATCCATACACAGGACCC -3′ (reverse); TM, 5′-TTGTGGAATTGGGAGCTTGG-3′ (forward), and 5′-TCTCATGAAC TGGATGGGGTG-3′ (reverse); and SIRT1, 5′-GCCAGAGTCCAAGTTTAGAAGA-3′ (forward), and 5′-CCATCAGTCCCAAATCCAG-3′ (reverse). To compare the mRNA levels of SIRT1, KLF2, TM, and eNOS among various cell lines, real-time PCR analysis was conducted using the ABI Prism 7500 Sequence Detection System with the EvaGreen Master Mix (Biogenesis, Taipei, Taiwan). PCR amplification consisted of an initial denaturation step (95 °C for 3 min) and 40 cycles of denaturation (95 °C for 15 s), annealing, and extension (60 °C for 1 min). The forward and reverse primers for the amplification of the cDNA were the same as those used in RT-PCR.

### 4.4. Western Blot Analysis

Cells were washed in PBS and isolated by scraping. Scraping was conducted on ice in a RIPA buffer and protease-inhibiting cocktail (Complete Roche Molecular Biochemicals, Almere, Netherlands). Western blot analysis was conducted on 20 µg total protein. Samples were run on 10% SDS-PAGE, electroblotted onto membranes, and incubated with primary antibodies against human eNOS (AP11828a; Abgent, San Diego, CA, USA), p-eNOS (612392; BD Biosciences, San Jose, CA, USA), TM (THBD, A4155; ABclonal, Woburn, MA, USA), SIRT1 (8469S; Cell Signaling Technology, Inc., Danvers, MA, USA), KLF2 (AP14973; Abgent, San Diego, CA, USA), and β-actin (A5441; Sigma-Aldrich, St. Louis, MO, USA). Proteins were detected with enhanced chemiluminescence using peroxidase-labeled luminol as the detection fluid (ECL; Amersham Life Sciences, Buckinghamshire, UK).

### 4.5. Functional Assay of TM

TM was measured by in situ endothelial cell culture in a 96-well plate with a designed concentration of cilostazol, according to our previous report [47]. After the removal of the culture medium from the cells followed by washing in serum-free EGM-2 medium, human protein C and thrombin (Enzyme Research Laboratories, Sketty, Swansea, UK) were added to each well to achieve a 150 μL total volume. Then, the cells were incubated at 37 °C for 2 h. Thrombin activity in each well was assessed by incubation for 5 min with 50 μL hirudin (50 U/mL) (CGP39393; Ciba-Geigy, Horsham, W. Sussex, UK), followed by the transfer of 200 μL supernatant from each well to a new microplate at 37 °C, and 50 μL chromogenic substrate S-2366 (82 1090 39; Chromogenix, Milano, Italy) was added. TM activity was measured using absorbance at 410 nm every 15 min by serum albumin at 37 °C. The absorbance change at 410 nm was measured with a ThermoMax Microplate Reader (Molecular Devices Corp., Sunnyvale, CA, USA). The wells containing thrombin and protein C in the absence of cells were used as a control.

### 4.6. Detection of NO by Flow Cytometry

Two million HUVECs were cultured in 3.5 cm dishes and treated with varying doses of cilostazol for 24 h. Then, the cells were stained with 1 µg/mL 4,5-diaminofluorescein (205391-01-1; Cayman Chemicals, Ann Arbor, MI, USA) for 30 min and harvested, followed by washing twice with PBS. After treatment, these cells were analyzed using flow cytometry (BD Biosciences, San Diego, CA, USA).

### 4.7. Genetic Knockdown Using the Lentivirus shRNA System

Transfection plasmids of short hairpin RNA (shRNA) for *KLF2*, *SIRT1*, *AKT1*, and *PRKAA2* were obtained from the National RNAi Core Facility located at the Institute of Molecular Biology/Genomic Research Center, Academia Sinica. Lentiviral particles were generated in 293T cells grown in DMEM supplemented with 10% FBS in an incubator at 37 °C and 5% CO_2_. Cells were plated to 2.4 × 10^6^ cells in a 10-cm dish before the transfection day. The 293T cells were transfected with 6.0 μg mock vector (shMock, shLuc, TRCN0000072243), shSIRT1 (TRCN0000229630), shAKT (TRCN0000199831), shPRKAA2 (TRCN0000002170), or shKLF2 (TRCN0000020725) with 5.4 μg pCMVDR8.91 and 0.6 μg pMD.G using virus *Trans*IT^®^-LT1 transfection reagent (MIR 2304; Mirus Bio, Madison, WI, USA), according to the manufacturer’s instructions. Then, recombinant lentivirus was prepared as stock, and the HUVECs were infected at a multiplicity of infection of 1–2. Transformants were selected with 2 μg/mL puromycin.

### 4.8. Statistical Analysis

The results were analyzed using the SPSS version 17 statistics software (SPSS Inc., Chicago, IL, USA). ANOVA and two-tailed Student’s *t*-test were used for comparisons. A *p*-value < 0.05 was indicated as statistically significant.

## 5. Conclusions

In summary, we revealed that cilostazol may potentially induce SIRT1 expression and then activate KLF2 transcription, which may result in increased eNOS and TM expression (Figure 8). These lead to NO and TM production, which could relate to thrombus formation. Activation of KLF2 also causes cell morphology changes, which occur because of cytoskeleton rearrangement. Our findings may provide a possible molecular mechanism by which cilostazol induces vasculoprotective and antithrombotic effects in endothelial cells.

## Data Availability

Data is contained within the article.
